# Evaluation of 3-Day Course of Doxycycline for the Treatment of Uncomplicated *Chlamydia trachomatis* Cervicitis

**DOI:** 10.1155/S1064744997000069

**Published:** 1997

**Authors:** Mark B. Reedy, Patricia J. Sulak, Sally L. Miller, Miriam Ortiz, Cheryl Kasberg-Preece, Thomas J. Kuehl

**Affiliations:** 1 Department of Obstetrics and Gynecology Scott & White Clinic 1600 University Drive East College Station TX 77840 USA; 2 Texas A&M University Health Sciences Center College of Medicne Temple TX USA; 3 Department of Biostatistics Scott & White Clinic Temple TX USA; 4 Department of Pathology Scott & White Clinic College Station and Temple TX USA; 5 Department of Medical Biochemistry and Genetics Scott & White Clinic College Station and Temple TX USA

## Abstract

*Objective:* The purpose of this study was to compare the efficacy of a 3-day course of doxycycline to a standard 7-day course for treating uncomplicated chlamydia cervicitis.

*Methods:* During an 18-month period, 77 women with uncomplicated chlamydia cervicitis were randomized to receive either a 3-day or a 7-day course of doxycycline (100 mg twice daily). Tests of cure were performed 3 weeks after completion of therapy with the Amplicor polymerase chain reaction (PCR) assay (Roche Molecular Systems, Branchburg, NJ). Demographics, therapeutic results, and side effects for the two groups were compared.

*Results:* Seventy-three patients completed the study: 35 in the 3-day group and 38 in the 7-day group. There were no significant differences in age, gravidity, or parity between the groups. There was a 94% (33/35) cure rate in the 3-day group and a 95% (36/38) cure rate in the 7-day group (*P* = 1.0). Thirty-four percent and 32% of the patients in the 3- and 7-day groups reported side effects, respectively; there was no significant differences between the 3- and 7-day groups in regard to population demographics, patient compliance, therapeutic outcome, or side effects.

*Conclusions:* A 3-day course of doxycycline appears to be as effective as a 7-day course of doxycycline for the treatment of uncomplicated chlamydia cervicitis.


Infectious Diseases in Obstetrics and Gynecology 5:18–22 (1997)
© 1997 Wiley-Liss, Inc.

### Evaluation of 3-Day Course of Doxycycline for the Treatment of Uncomplicated *Chlamydia trachomatis* Cervicitis

**Mark B. Reedy,[1,2*] Patricia J. Sulak,[1] Sally L. Miller,[1] Miriam Ortiz,[1] Cheryl Kasberg-Preece,[3] and Thomas J. Kuehl[1,4,5]**

[1]*Department of Obstetrics and Gynecology, Scott & White Clinic, College Station and Temple, TX*
[2]*Texas A&M University Health Sciences Center, College of Medicine, Temple, TX*
[3]*Department of Biostatistics, Scott & White Clinic, Temple, TX*
[4]*Department of Pathology, Scott & White Clinic, College Station and Temple, TX*
[5]*Department of Medical Biochemistry and Genetics, Scott & White Clinic, College Station and Temple, TX*

### Abstract

*Objective:* The purpose of this study was to compare the efficacy of a 3-day course of doxycycline to a standard 7-day course for treating uncomplicated chlamydia cervicitis.

*Methods:* During an 18-month period, 77 women with uncomplicated chlamydia cervicitis were randomized to receive either a 3-day or a 7-day course of doxycycline (100 mg twice daily). Tests of cure were performed 3 weeks after completion of therapy with the Amplicor polymerase chain reaction (PCR) assay (Roche Molecular Systems, Branchburg, NJ). Demographics, therapeutic results, and side effects for the two groups were compared.

*Results:* Seventy-three patients completed the study: 35 in the 3-day group and 38 in the 7-day group. There were no significant differences in age, gravidity, or parity between the groups. There was a 94% (33/35) cure rate in the 3-day group and a 95% (36/38) cure rate in the 7-day group ($P = 1.0$). Thirty-four percent and 32% of the patients in the 3- and 7-day groups reported side effects, respectively; there was no significant differences between the 3- and 7-day groups in regard to population demographics, patient compliance, therapeutic outcome, or side effects.

*Conclusions:* A 3-day course of doxycycline appears to be as effective as a 7-day course of doxycycline for the treatment of uncomplicated chlamydia cervicitis. Infect. Dis. Obstet. Gynecol. 5:18–22, 1997. © 1997 Wiley-Liss, Inc.

Key words
chlamydia; doxycycline; cervicitis; sexually transmitted diseases

*Chlamydia trachomatis* is the most common reportable sexually transmitted disease in the United States, with 3–5 million new cases estimated each year. The majority of genital chlamydial infections are asymptomatic in women but can result in pelvic inflammatory disease, ectopic pregnancy, and infertility.[1] Research on chlamydial infections has been concentrated on diagnostic testing rather than development of new therapeutic options mainly because no significant antimicrobial

Presented at the 1996 ACOG Annual Meeting, Denver, CO.
Contract grant sponsor: Scott, Sherwood, Brindley Foundation, Temple, TX.
*Correspondence to: Dr. Mark B. Reedy, Department of Obstetrics and Gynecology, Scott & White Clinic, 1600 University Drive East, College Station, TX 77840.

Received 20 September 1996
Accepted 3 March 1997

**TABLE 1. Exclusion criteria**

| |
|---|
| Pregnancy |
| Fever (>100.4°F) |
| Uterine or adnexal tenderness or a pelvic mass noted on examination |
| <18 years of age without consent of a parent or guardian |
| Antibiotic treatment of any kind since positive chlamydia test |
| Allergy to tetracycline or doxycycline |
| Patients attempting to conceive or using an unreliable form of birth control |
| Patients who cannot be easily contacted for follow-up (i.e., do not have a home or work telephone number) |
| Currently using an intrauterine device for birth control |

resistance has been demonstrated against standard treatment—the tetracyclines.

The 1993 Center for Disease Control (CDC) recommendation for the treatment of chlamydial cervicitis is 100 mg of doxycycline given orally twice daily for 7 days or azithromycin (Zithromax®, Pfizer, Groton, CT) 1 g orally in a single dose.[2] Currently, the authors' clinic pharmacy charges $25.90 for 1 g of azithromycin and $5.30 for a 7-day course of doxycycline.

In 1985, the CDC stated that the treatment recommendation for chlamydia had a considerable potential for patient noncompliance.[3] Cramer et al.[4] demonstrated that the level of compliance deteriorates the longer the course of therapy. A shorter therapeutic regimen would improve patient compliance and be more cost effective. This was recently demonstrated in the treatment of urinary tract infections.[5]

The purpose of this study was to compare a 3-day course of doxycycline to a 7-day course for the treatment of uncomplicated chlamydia cervicitis.

### SUBJECTS AND METHODS

A prospective, randomized, open-labeled study was performed. Nonpregnant women in a multispecialty clinical setting with cervical swabs positive for chlamydia by polymerase chain reaction (PCR) assay (Amplicor PCR®, Roche Molecular Systems, Branchburg, NJ) were eligible for the study. Parental consent was required for patients under 18 years of age. Study enrollment required the patient to be afebrile and have a normal pelvic examination. Informed consent was obtained. Exclusion criteria are listed in Table 1.

A total of 77 nonpregnant women with uncomplicated *Chlamydia trachomatis* cervicitis were randomized into 2 treatment arms. Group 1 received the standard CDC-recommended treatment consisting of 100 mg doxycycline twice daily for 7 days. Group 2 received 100 mg doxycycline twice daily for 3 days. A central office allocated patients by computer-generated randomization schedule.

Patients were treated free of charge. Sexual partners were also offered the standard 7-day course of doxycycline free of charge. Patients were asked to refrain from intercourse with their partner until they had completed their medication. If the patient missed a menstrual cycle or was not practicing a reliable form of contraception (i.e., oral contraceptives, tubal ligation, Norplant®, Depo-Provera®), a serum pregnancy test was performed. If a coexisting cervical infection with *Neisseria gonorrhoeae* was present, patients were treated with 250 mg of intramuscular ceftriaxone.

Each patient returned approximately 3 weeks after completion of their medication. At the follow-up appointment, a cervical swab was obtained for chlamydia PCR assay, and each patient was asked questions from a post-treatment questionnaire. Patients received a $30.00 stipend when they returned for the follow-up visit.

The post-treatment questionnaire consisted of the following questions: 1) How many pills did you take? 2) Did you have any side effects, i.e., vomiting, headache, diarrhea, stomachache, and did you take the medicine with food? 3) Did your partner take all of his medicine? If not, how many pills did he take? 4) Did you have intercourse with your partner or anyone else since your enrollment into the study?

All data forms and questionnaires were forwarded to a central site to be inventoried and entered into an established data management system. Data collection forms were checked for inconsistencies and missing values by a computerized edit-checking system. Any inconsistencies were reported to the investigator for correction or verification. Summary and descriptive statistics for all baseline variables were performed using SAS software package (Cary, NC). Comparisons between study groups were done using the Student's t-test or chi-square analysis on the appropriate demographic variables. Adherence to the prescribed treatment within each group was assessed by performing pill counts. Incidence of side effects was

TABLE 2. Description of 4 treatment failures

| Regimen | Age (years) | Gravidity/parity | All medicine taken? | Side effects? | Medicine with food? | Partner take all medicine? | Intercourse? | Follow-up (weeks) |
|---|---|---|---|---|---|---|---|---|
| 3 day | 19 | 0/0 | Yes | No | Yes | Yes | Yes | 2.0 |
| | 14 | 0/0 | No (only 5) | No | No | No (only 2) | Yes | 3.1 |
| 7 day | 16 | 0/0 | Yes | No | Yes | No (none) | Yes | 7.1 |
| | 30 | 0/0 | Yes | No | Yes | Yes | No | 3.0 |

compared between groups by chi-square analysis or Fisher's exact test, as appropriate. The primary outcome, efficacy, was defined as the number of negative tests of cure per group divided by the total number of patients who completed the study treatment. These proportions were analyzed using chi-square test.

Power analysis performed prior to the study determined that a sample size of 35 patients in each group would detect a 25% difference in efficacy of cure with 80% power and a 0.05 level of significance.

### RESULTS

During an 18-month interval, 77 women were randomized to receive either a 3- or 7-day course of doxycycline. Three patients were lost to follow-up (1 patient in the 7-day group and 2 patients in the 3-day group). Another patient was dropped from the study after randomization when a repeated pregnancy test was positive. Seventy-three patients completed the study. Thirty-five patients were randomized to the 3-day group and 38 patients to the 7-day group. The mean age of the 3-day group was 21.8 (±5.5 years), compared with 21.3 (±4.5 years) in the 7-day group. There was no significant differences among groups in age ($P = 0.65$), gravidity ($P = 0.16$), or parity ($P = 0.09$).

Follow-up PCR assays were obtained approximately 3 weeks after completion of therapy. There was no significant difference ($P = 0.27$) in follow-up intervals between the 3-day (3.7 weeks) and the 7-day (4.2 weeks) groups. Of the 35 patients in the 3-day group, there were 2 treatment failures (efficacy, 94%). The 7-day group had 2 treatment failures out of 38 patients (efficacy, 95%). There was no significant difference in efficacy between the two groups (Fisher's exact test $P = 1.0$). Table 2 provides descriptions of the 4 treatment failures.

Patients in the 7-day group took all their medication appropriately. Two patients in the 3-day group did not complete their medication. One patient took only 5 pills over a 3-week period instead of 3 days in a row. This patient's test of cure was positive. The second patient took 4 pills over 2 days and did not take the 3rd day of pills due to nausea. This patient's test of cure was negative.

TABLE 3. Side-effect profile

| Side effect | 3 day | % | 7 day | % |
|---|---|---|---|---|
| None | 23 | 66 | 26 | 68 |
| Nausea | 8 | 23 | 8 | 21 |
| Vomiting | 5 | 14 | 3 | 8 |
| Stomach upset | 2 | 6 | 3 | 8 |
| Headache | 2 | 6 | 3 | 8 |
| Diarrhea | 1 | 3 | 2 | 5 |

Thirty-four percent of patients in the 3-day group and 32% in the 7-day group reported side effects ($P = 1.0$). The most common side effect was nausea (Table 3).

### DISCUSSION

*Chlamydia trachomatis* has a complex and long life cycle. The elementary body (infectious particle) attaches to and enters the endocervical cells. The elementary body remains as a cytoplasmic inclusion throughout its life cycle within the cell. Within the inclusion body, noninfectious but metabolically active reticulate bodies are formed. The reticulate bodies condense to form elementary bodies. The typical life cycle takes between 48 and 72 h to complete. For an antimicrobial agent to be effective against chlamydia, it must be present over a period of 2–3 days.[6]

Azithromycin is a new macrolide antibiotic which has been shown to be as effective as a 7-day course of doxycycline in the treatment of uncomplicated chlamydia infections of the uterine cervix. Azithromycin is effective in treating chlamydia with a single dose due to its long half-life (68 h) and adequate tissue penetration.[6]

Doxycycline is one of the current drugs of choice for the treatment of chlamydia cervicitis. It is the drug to which all other therapeutic regimens are compared. It is well absorbed by the gastrointestinal tract within 1–3 h and food does not impair absorption. The serum half-life is 12–18 h after a single dose and 18–22 h after several doses. A single 200 mg oral dose achieves serum levels of 2.5 µg/ml and intravenous administration offers no benefit over oral dosing. The mean inhibitory concentration (MIC) of chlamydia is 0.06 µg/ml. Doxycycline is effectively distributed to the female genital tract.[7] It is one of the most active agents against chlamydia as demonstrated by in vitro susceptibility testing and human clinical trials.[8] A large body of literature is available which demonstrates that a 7-day course of doxycycline has a failure rate of 0–8% for uncomplicated chlamydia cervicitis.[9]

A few reports have shown that shorter dosing regimens of antibiotics are as effective in treating chlamydia as the standard 7-day course of doxycycline or erythromycin. Stamm et al.[10] compared a 3-day course of trimethoprin-sulfamethoxazole (TMP-SMZ) with a 5-day course of tetracycline and found 30 of 32 (94%) women cured in the TMP-SMZ group and 27 of 29 (93%) cured in the tetracycline group. Bowie et al.[11] compared the efficacy of several treatment regimens for lower genital tract chlamydia infections in women. They made an interesting observation that of the 3 women who took only 3 days of ampicillin, all had negative cultures. Also of interest is a study by Levallois and Rioux[12] in which they performed a randomized, double-blind, placebo-controlled trial of doxycycline for suction curettage abortion prophylaxis. Seventy-five women who preoperatively screened positive for chlamydia were randomized into a prophylaxis and placebo group. Prophylaxis consisted of 100 mg of doxycycline preoperatively and a 200 mg dose postoperatively. In the prophylaxis group, only 1 of 33 (3.0%) had signs or symptoms of post-abortal pelvic infection vs. 11 of 42 (26.2%) of women in the placebo group. Though this study's endpoint was post-abortal pelvic infection, a 1-day course of doxycycline significantly reduced the risk of infection in women with chlamydia infection.

We elected to perform a test of cure 3 weeks following the completion of therapy. We chose 3 weeks instead of a longer time interval for the following reasons. First, we were concerned that the longer the interval from completion of therapy to test of cure, the greater the risk of reinfection and losing the patient for follow-up.[2,4,6] Second, after reviewing the literature, we found that many studies, including the recommendations from the CDC, use between 2 and 4 weeks for repeat testing.[2,13–16] Workowski et al.[16] studied the duration of detecting chlamydia by PCR and tissue culture following a 7-day course of doxycycline. They found that chlamydia is eradicated immediately after treatment as detected by tissue culture, but can be detected up to 1 week following therapy by PCR.

The major side effects of doxycycline in patients are related to gastrointestinal effects. We anticipated that shortening the course of therapy would reduce the side effects. However, there was no significant reduction in side effects when compared to the 7-day course of therapy. We believe this demonstrates that side effects from doxycycline occur early in the course of therapy. Though there was no difference in patient compliance in this study, we believe patients off study protocols are more likely to complete a 3-day course of therapy than 7 days.

This study was designed as a preliminary study to see if a 3-day course of doxycycline was a feasible treatment option for chlamydia cervicitis. Therefore, we chose a study design which could identify a 25% difference between groups at a power of 0.8 and a $P$-value of 0.05. For comparison, it would take 280 patients (140 per treatment arm) to perform a study which could identify a 10% difference between groups with a power of 0.8 and a $P$-value of 0.05. Our institution does not have a sexually transmitted disease population large enough to perform a study of this magnitude in a reasonable amount of time. We believed that if no statistical difference was found between a 3- and a 7-day course of therapy, further study would be warranted.

There were no statistically significant differences between groups in population demographics, patient compliance, efficacy, or side effects when comparing the 3-day and the 7-day study groups. A 3-day course of doxycycline appears to be as effective as a 7-day course of therapy for uncomplicated chlamydia cervicitis. This report provides strong preliminary data to support further investigation with this treatment regimen.

### REFERENCES

1. Sweet RL, Gibbs RS: Chlamydial infections. In: Sweet RL, Gibbs RS: Infectious Diseases of the Female Genital Tract. 2nd ed. Baltimore: Williams & Wilkins, pp 45–74, 1990.
2. Centers for Disease Control and Prevention: 1993 sexually transmitted disease guidelines. MMWR 42(RR-14): 1–102, 1993.
3. Centers for Disease Control: *Chlamydia trachomatis* infections: Policy guidelines for prevention and control. MMWR 34(Suppl 3s), 53s–74s, 1985.
4. Cramer JA, Scheyer RD, Mattson RH: Compliance declines between clinic visits. Arch Intern Med 150:1509–1510, 1990.
5. Trieneken TAM, et al.: Different lengths of treatment with co-trimoxazole for acute uncomplicated urinary tract infections in women. Br Med J 299:1319, 1989.
6. Stamm WE: Azithromycin in the treatment of uncomplicated genital chlamydia infections. Am J Med Suppl 3A:195–225, 1991.
7. Cunha BA, Comer JB, Jonas M: The tetracyclines. Med Clin North Am 66:293–301, 1982.
8. Martens MG, Faro S, Maccato M, Riddle G, Hammill H, Wang Y: In vitro susceptibility testing of clinical isolates of *Chlamydia trachomatis*. Infect Dis Obstet Gynecol 1:40–45, 1993.
9. Centers for Disease Control: Recommendations for the prevention and management of *Chlamydia trachomatis* infections. MMWR 42(RR-12):27–28, 1993.
10. Stamm WE, Guinan ME, Johnson C, Starcher T, Holmes KK, McCormack W: Effects of Treatment Regimens for *Neisseria gonorrhoeae* on simultaneous infection with *Chlamydia trachomatis*. N Engl J Med 310:545–549, 1984.
11. Bowie WR, Manzon LM, Borrie-Hume CJ, Fawcett A, Jones HD: Efficacy of treatment regimens for lower urogenital *Chlamydia trachomatis* infection in women. Am J Obstet Gynecol 142:125–129, 1982.
12. Levallois P, Rioux J-E: Prophylactic antibiotics for suction curettage abortion: Results of a clinical controlled trial. Am J Obstet Gynecol 158:100–105, 1988.
13. Steingrimsson O, Olafsson JH, Thorarinsson H, Ryan RW, Johnson RB, Tilton RC: Azithromycin in the treatment of sexually transmitted disease. J Antimicrob Chemother 25(Suppl A):109–114, 1990.
14. Martin DH, Mroczkowski TF, Dalu ZA, McCarty J, Jones RB, Hopkins SJ, Johnson RB: A controlled trial of a single dose of azithromycin for the treatment of chlamydia urethritis and cervicitis. The Azithromycin for Chlamydial Infection Study Group. N Engl J Med 327: 921–925, 1992.
15. Hooten TM, Batteiger BE, Judson FN, Spruance SL, Stamm WE: Ofloxacin versus doxycycline for treatment of cervical infection with *Chlamydia trachomatis*. Antimicrob Agents Chemother 36:1144–1146, 1992.
16. Workowski KA, Lampe MF, Wong KG, Watts MB, Stamm WE: Long-term eradication of *Chlamydia trachomatis* genital infection after antimicrobial therapy. Evidence against persistent infection. JAMA 270:2071–2075, 1993.
